# Interdisciplinary Perspectives on Breath, Body and World

**DOI:** 10.1177/1357034X20913103

**Published:** 2020-04-27

**Authors:** Rebecca Oxley, Andrew Russell

**Affiliations:** Durham University; Durham University

**Keywords:** body, breath, ethnography, interdisciplinarity, world

## Abstract

Breath, the ephemeral materialization of air at the interface of body and world, engages with and alters the quality of both. As a process of inhalation and exhalation that signals its physiological universality, breath is an invisible prerequisite for life, an automated and functional necessity. Yet it is more than simply a reflexive action and can at times be controlled or manipulated. It can also affect or be affected by experiences, environments and relationships. In this essay, like the contributors to the special issue it prefaces, we aim to address the lacuna that exists in the examination of the meanings and embodiment of breath as a central theme in the humanitics and social sciences. Interdisciplinary perspectives that explore breath as a multifaceted phenomenon, both intrinsically shared and contextually distinct, open new directions in the field of breath and body studies.

## Breath, Body, World – Exploring the Relations

Breath is the ephemeral materialization of air at the interface of body and world, engaging with and altering the qualities of both. It is most commonly understood in terms of the process of inhalation and exhalation that signals its physiological universality. In this mode of understanding, breath is an invisible precedent for life, an automated and functional necessity. Yet breath is more than simply a reflexive action, not least because it can be controlled or manipulated at times, and can affect or be affected by experiences, environments and relationships. Despite the merits of exploring breath as a phenomenon that is at once intrinsically shared but contextually distinct, the meanings and embodiment of breath has seldom been examined as a central research theme in the humanities and social sciences.

One of the exceptions is the work of Irigaray (1999, 2002, 2004). She questions conventional scholarly traditions to propose an ontology of breath as a means to explore the grounds where consciousness and body meet. This offer has been extended by Škof and Holmes (2013), suggesting how breath can be a lens to connect dualisms such as self and other, body and spirit, and East and West. More recently, Górska (2016) has proposed a non-universalizing, intersectional consideration of embodiment through approaching breath as a material discursive enactment or, more specifically, how bodies enact politics through breathing. Lande’s (2007) work on breathing like a soldier has also provided ethnographic inquiry into how culture can become embodied through the breathing body in the military milieu. However, discussions of breath, and its significance for the field of body studies, usually arise indirectly, as an aligned interest, or as an emergent motif – even when such insights are significant, as with Ingold’s (2010) consideration of breath, knowledge and movement in the weather-world. There are similarly rich understandings to be gleaned from material on cosmology (e.g. Long, 1996; Mann, 2016), airs or winds (e.g. Hsu, 2007; Yoeli-Tlalim, 2010), yogic practice (e.g. Smith, 2007), ritual healing (e.g. Brabec de Mori, 2015), singing/musical performance (e.g. Harris, 2016) or illness (e.g. Carel, 2008). Yet what comprises breath and its experience remains primarily the terrain of the clinical, medical or public health sciences.

The central premise of this review, and of the special issue articles to which it is a prelude, is that breath needs to be understood in broader terms than the clinical, as a mode of relating to the world, engaging with others, objects, environments and technologies. These relations are fluid but have patterns and rhythms; breath can frame the pace of daily life and experience as well as being the nebulous essence of being-in-the-world. Crucially, the significance and meaning of breath varies according to the contexts in which it is appraised and enacted. In this introductory essay, we aim to explore some of these contexts using appropriate ethnographic and historical materials. It derives from research conducted by Durham and Bristol Universities through the Wellcome Trust-funded *Life of Breath* project (http://lifeofbreath.org/), a collaborative, interdisciplinary programme exploring breathing and breathlessness at the interface between arts, humanities, social sciences and clinical practice (Macnaughton and Carel, 2016). Taking breathing bodies and environs as a holistic cue, we review some texts that engage with the embodied meanings of breath and, like breath itself, complicate dualisms such as inside/outside, absence/presence and appearance/disappearance. We hope our essay, like the special issue it introduces, counteracts the serious lacuna of breath in body studies, where breath has too often been ignored, invisible or taken for granted.

## Breath, Its Origins and Vitality

Breathing arguably signals the first independent and autonomous act of life. Yet, for many ontologies, breath is not only fundamental for individual life but can be the very basis of what comprises a more amorphous, yet frequently powerful, collective experience. Certain foundational works in social anthropology offer insights into some of the intricacies of breath as a vital force of life and health and the multiple meanings and embodied experience it offers people, body and world. Some of the wider themes that emerge throughout this review are already apparent in texts offered by Bronsilaw Malinowski, Marcel Mauss and Sir James George Frazer. For Malinowski, the notion of the ‘breath of life’ was considered one of his key ethnographic motivators. In *Argonauts of the Western Pacific*, he writes:

What interests me really in the study of the native is his outlook on things, his *Weltanschauung*, the breath of life and reality which he breathes and by which he lives. Every human culture gives its members a definite vision of the world, a definite zest of life. In the roamings over human history, and over the surface of the earth, it is the possibility of seeing life and the world from the various angles, peculiar to each culture, that has always charmed me most, and inspired me with real desire to penetrate other cultures, to understand other types of life. (Malinowski, [1922]2014: 534)

Malinowski uses the phrase the ‘breath of life’ in terms of a general world view and as an essential indicator of being, rather than in a specified cosmological sense, but already the latent threads between the phenomena of breath and appreciating lived experience are apparent.^1^

The subject of breath in early anthropological work seems to manifest as a leitmotif of magic, the taboo and shamanistic power. For Mauss however, comprehending breath forms the basis of his ‘general theory of obligation’. In *The Gift* ([1950]1990), Mauss looked to the Māori concept of *hau*, which has many complex and subtle meanings including breath, vitality and remittance, as the ‘spirit’ of the gift. However, Mauss’ reading of *hau* has encountered critique at its seemingly narrow interpretation, and its dissociation from Māori ontologies and their knowledge of the ‘breath of life’ (Thompson, 1987). As such, it provides a clear example of the importance of grasping phenomena within their wider cultural milieu. A more apparent, and more recent example of such misrepresentation was raised by Barbara Mann in *Spirits of Blood, Spirits of Breath: The Twinned Cosmos of Indigenous America* (2016). Mann details how certain Indian cultures of Turtle Island (pre-invasion North America) held a binary belief of a twinned cosmos; half blood, half breath, that was misconstrued into a notion of the ‘sky’ and ‘earth’ fraught with Christian overtones. In this sense, the spirit ‘Master of Breath’, an intrinsic part of the creation of life in Cree mythology, became the ‘Great Spirit’ or ‘Almighty Spirit’ and supreme giver-of-life close to the Christian God (p. 196).

In Western Jewish and Christian traditions, the Almighty Spirit of God is the source of all life, breath and inspiration, with the Hebrew term *ruach* indicating the spirit, wind and breath of God (Škof and Holmes: 8). It is *ruach* which provides life to mankind when ‘the Lord God formed man of dust from the ground, and breathed into his nostrils the breath of life; and man became a living being’ (Gen. 2.7). While not exactly comparable to *ruach*, for the Khoisan peoples of southern Africa, the wind *as* the breath of God gives life. Writing as part of a special edition of the *Journal of the Royal Anthropological Institute* on the experience and phenomenology of wind, Chris Low (2007) details how the Khoisan mirrored or reproduced elements of Christianity to thread together their understandings of divine creation. He writes:

In wind, God bestows and unites life and removes the footprints of the departed from the dust. The idea of breathing in a life-giving, God-derived wind overlaps with notions of soul. A number of Nama women explained to me that when we breathe in and out we are breathing in and out */om*, soul. It is the wind that works with the heart. In Khoekhoegowab (mainly Nama, Damara, Hai//om) the same word, */om*, is used for breath and soul. The wind that God gives people and animals is a specific gift of life. The gift is a personal wind that underlies an organism’s form and action and constitutes its potency. The gift of life is visible in the breathing, standing, and participation of people and animals in a shared environment. (S74)

This ideology of wind as evidence of divinity also pertains to the Mayan peoples living by the Main Chasm, Aguateca, Guatemala, in the late classic period. As caves were viewed as animate entities, wind flowing around cave openings not only proved that caves could breathe but confirmed the presence of the Anhel (a´ngel), the Tzotzil rain deity who resided in caves (Ishihara, 2008: 177).

Low’s work with the Khoisan also introduces the notion that breath, soul and spirit are often correlated in understandings of life. Echoing Lyall Watson’s (1999: 5) popular suggestion that ‘[T]he ideas of life and breath and spirit and smell are intertwined in many cultures’, David Parkin (2007) proposes that a useful method to conceive of this correlation may be to consider a semantic template of ‘airiness’. He compares Indo-European, and Jewish-Arabian terms with those of Bantu speakers of eastern and central Africa to agree with Watson’s popular suggestion that *smell* is of key importance.^2^ Parkin’s idea of a semantic template is useful when thinking of ‘the breath of life’ as pertains to Ancient Egyptian mythology. In *The Golden Bough: A study in Magic and Religion* ([1922] 2000), Frazer notes that Isis, the goddess of magic, life and welfare held the breath of life and the power to heal. It was Isis who resurrected Osiris and instructed the life-giving Nile to rise each year. However, this power was not exclusive to Isis, but one which was shared among other deities including Amum and the god-king Pharaohs. Life and divinity could be bestowed upon images, statues and mummies through the breathing of vital incense upon them. Incense, symbolizing the sweat of Amum that wafts life upon the gods, transferred the ‘odor of the living body’ into inanimate beings (Wise, 2009: 72). Incense (from the 19th dynasty onwards) was thus a significant part of the ‘opening of the mouth ceremony’, that enabled mummies to speak and to breathe (p. 74).

While these examples detail divine breath as infusing humanity or sentient life and (re)vitalizing the lifeless, the work of the Ancient Greek Stoics suggests an understanding of the breath of god pervading the whole material world. *Pneuma*, as vital heat or air in motion, the breath of Fire the Craftsman (Zeus), held various functions and manifestations (*physis, psyche*) that provided a cohesive property for stones, plants, animals and human beings. People, imbued with a rational soul that set them apart from other material entities, were considered ‘intelligent warm breaths’ (Long, 1996). These cross-cultural understandings of the ‘breath of life’ are rare – and particularly so with regard to classical texts – yet they offer a potentially crucial insight not only into the fine-tuned complexities of comprehending the origin of life and its categorization, but also the intricate and often unexplored relationship between breath, spirit, soul and wind.

## The Final Breath

The Dinka seem to capture the vitality of their ritual clan chief, the ‘master of the fishing spear’, as death approaches. Lienhardt describes a ceremony, *dhor beny ke pir*, which he translates as ‘to bury a master while he lives’. People seek to

*dhor* the clan-divinity so that it may augment their strength, and in doing so satisfy a demand which it makes upon them. Similarly…in placing their master of the fishing spear in the grave while he yet lives, they think to augment their vitality and also, normally, to gratify his own desire. (Lienhardt, 1961: 299)

If breath is an essence of life, it can also be an important marker of death and passage into the afterlife. The notion of soul as an embodied spirit that leaves the body after one’s final breath is common to a number of belief systems. Simon Rae, in his study of the Karo people of the highlands of North Sumatra, details that the most important and pressing aspect of Karo religion occurs with consideration of the *begu*, the soul or *tendi* of departed kin and ancestors (1994: 19). This is indicated in the customary proverb:

*tendi jadi begu* the soul becomes a spirit

*buk jadi ijuk* the hair becomes a course black fibre

*jukut jadi taneh* flesh becomes earth

*tulan jadi batu* the bones become stones

*dareh jadi lau* the blood becomes water

*kesah jadi angin* the breath becomes the wind (p. 19)

For the Karo, understandings of spirit are not conceived as in opposition to the material body, as might be considered in the Cartesian tradition. Vishvajit Pandya (2007) perceives that questioning such conventional Western modes of envisioning breath and soul is critical to comprehending modes of life in the Andaman Islands. He notes:

For [the West] there is an ontological opposition between spirits and matter…[This] opposition implies that spirits are immaterial. Their substance is ‘breath’, most invisible in the visible, most immaterial in the material. Spiritual existence is understood as the existence of an animating principle without a body or outside body. But no spirit in the Andamanese world has this disembodied existence, nor is it reducible to such an existence. This is exemplified by the fact that the closeness of spirits causes a shivering of the human body. (2007: S95)

However, as Parkin elaborates, for the people of the East African coast, breath can also be that which influences human reaction such as shivering, particularly as the movement of air or wind as spiritual breath can suggests the manifestation of spirits (2007: 40). In other words, for the Andamanese and those from the east coast of Africa, the substance of breath and its effects can be the very actualization of the spirit form but this can only occur in the context of interaction.

This realization extends to evidence of spirits accessed via scientific methodologies. Early 20th-century Massachusetts doctor Duncan MacDougall attempted to capture the discarnate human spirit/soul scientifically by measuring tuberculosis patients in an old age home after they exhaled their final breath. The soul, he concluded, had mass: it weighs ‘three-fourths of an ounce’, or 21 grams (Drageset, 2013: 10). This ‘escape’ of the soul at the moment that breath stops is one which has been deeply contemplated across the globe and has led to various techniques to seek to keep the soul within the body either to maintain life or to confine the spirit. Frazer ([1922] 2000) details how for both the Marquesans and New Caledonians, it was common practice to hold the nose and mouth of those dying, to continue their life by preventing their souls from departing. He also noted that the Nia people, who identify spirits of the recently deceased with breath, thought to bind the soul to the body by tying the jaw and blocking the nasal cavity. These techniques, while contextually specific, may be widely comparable to the difficult moral and ethical considerations surrounding those utilizing life support technologies in hospital or hospice settings. For those who understand the spirit as an animating principle, the lack of breath can be tantamount to the lack of an embodied self.

## Breath in Human Experience, Health and Illness

Breath is a key concept with which to understand the lived reality and substance of soul and spirit, and in doing so can be significant to reveal what it means to be alive or to be human. For the Kukajata people of the Australian western desert, to breathe and to live is to acknowledge a shared history with the environment as ‘the wind, both material and spiritual, becomes breath as it penetrates the body’s opening. As breath, the wind in not conceived of as intrusive, given that “the wind is thus cosubstantial with humans; they share the same ancestral essence”’ (Poirier, 2004, cited in Parkin 2007: S40).

This understanding of the human body as embodied spiritual nature is also one that pertains in a sense to the ancient Indian people from the time of the *Rgyveda* (c. 1500–1000 BCE) to the *Upanisads* (c. 800–500 BCE). Indians across this period also perceived the natural wind (*vāta* or *vāyu*) and breath in living beings (*prāna*) to be of the same essence. This belief informed a complex doctrine of the human body as comprised of different types of bodily winds. As noted by Kenneth Zysk (2007) in his account of ancient Indian bodily winds, two *Upanisads* offer early efforts to identify and locate five winds and their roles within the body:

*Praśna Upanisad* (3.5-7) provides their locations:

Prāna is located in the eyes, ears, mouth, and nose.

*Apāna* is located in the organs of excretion and generation.

*Samāna* is located in the middle of the body and distributes digested food.

*Vyāna* is located in the numerous channels of the body, all of which originate from the region of the heart.

*Udāna* rises up from one of the central channels and functions in moral actions.

The *Maitrī Upanisad* (2.6) gives their functions:

*Prāna* is the wind that passes upward.

*Apāna* is the wind that passes downward.

*Vyāna* is the wind that supports *prāna and apāna*.

*Samāna*, being a higher form of *vyāna*, is the wind that brings the coarse element of food into the *apāna* and distributes food’s subtle elements in the limbs.

*Udāna* is the wind that is between the *vyāna* and *samāna*. It belches forth and swallows down what is drunk and eaten. (S107)

Body knowledge as represented in the *Upanisad*s is highly detailed and complicated but seem to promote the notion that health is the state in which there is a natural or balanced flow of bodily winds. This is particularly elaborated on through the description of nasal breath and its relation to particular nostril modes and nerves (*Nadis*) introduced with the *Svara-udaya* (Nair, 2007: 88). Breath in these understandings constitutes the motion that combines body with mind, in which dwells the elemental soul. It therefore correlates with the ideology that *prānāyāmā* (breath control) is one key to *Brahman* or eternal consciousness. *Brahman* is the meditative state to which humanity should strive.

For the Javanese, breath control is also interlinked with what it means to be alive and well, but also, in a wider sense, to identify as human. In Java, Clifford Geertz describes how local people say that ‘to be human is to be Javanese’ (1973: 52). He elaborates that this simple phrase signifies the highly elaborate system of etiquette of the Javanese people, whereupon small children, the blatantly immoral and those living with mental illness or learning disabilities would be considered *ndurung djawa*, or ‘not yet Javanese’. Those adults who were *insampun djawa* or ‘already Javanese’ would be those with the ability to truly appreciate the subtleties of music, drama, dance and textile design, as well as those gifted with spiritual introspection (pp. 52–53). Geertz continues, that for the Javanese ‘to be human is not just to breathe; it is to control one’s breathing, by yogalike techniques, so as to hear in inhalation and exhalation the literal voice of God pronouncing His own name – ‘hu Allah’’(p. 53). To be human for the Javanese is thus a normative process utilizing breathing techniques to aim for health as conscious, spiritual awareness.

These understandings of body as breath/wind and of humanity as conscious breathing imply that health as with breath has a rhythm that must be constantly managed and worked on. In other words, vital health or humanity can be experienced only at the point of embodied realization. This differs slightly in inference from certain phenomenological understandings of health as going somatically unnoticed, only to be defined in relation to emerging conscious awareness of a threat to health such as with illness or disease. It is this understanding of health which gave rise to Drew Leder’s concept of *dys-appearance*, drawing upon Martin Heidegger’s analysis of the tool, to conceive of the moment where the body appears in thematic focus in a dysfunctional state (1990: 84). This moment of sickness or risk realization has informed the influential notion of ‘biographical disruption’, or chronic illness being considered a ‘disruptive event’ or ‘critical situation’ (Bury, 1982). This is particularly important considering that biographical disruption has been a key means to explore and comprehend the lived experience of chronic obstructive pulmonary disease (COPD) in western biomedical settings (e.g. Habraken et al., 2008; Hansen et al., 2007; Jownsey et al., 2014; Luz and Basto, 2013; Thorns and Cawley, 2011). It is this apparent ‘forgetting of air’ that prompts Luce Irigaray to look to Eastern traditions and modes of embodied experience that prioritize the cultivation of breathing (2002, 2013).

Breath then has a role to play in highlighting important frameworks through which a range of illnesses may be experienced and interpreted both temporally and spatially. This may especially be the case in terms of respiratory illnesses regarded as culture bound syndromes, such as ‘howler-monkey sickness’ for the Warao of Venezuela reconceived as whooping cough, the sensation of being ‘hit by the wind’ in Vietnam, causing hyper-vigilance and breathlessness, and the historical and multifarious ‘rising of the lights’ commonly associated with croup (Cock, 1926; Hinton et al., 2003: 343, 344; Olsen, 1996: 158; Taylor, 1926). The question thus can also be raised as to the place of traditional versus modern biomedical therapies when considering such sicknesses. Ronit Yoeli-Tlalim contemplates how the West might learn from cross-cultural notions of the body, sickness and healing by describing concepts of culture-bound ‘wind illness’ in Tibetan and Buddhist medical practice (2010). She describes how Tibetan understandings of health relate to a balanced flow of five bodily winds indicated in the *Four Tantras* (life-sustaining, ascending, pervading, fire-like, and descending winds), where risk to or cause of illness are associated with the three *nyes pas* of *rhlung* (wind), *mkris pa* (bile), and *bad kan* (phlegm) (pp. 320–321). Given that experience is considered to fall along a continuum, with spiritual attainment at one end and critical ill health at the other, the focus of healthcare is on restoring health and preventing illness through interventions to lifestyles, breathing practices and medicine (pp. 321–322).

Yoeli-Tlalim also acknowledges similarities between this outlook to understandings of the Greek *pneuma*, and the Chinese notion of *qi*. Tadashi Ogawa (1998) details how *qi* or *ki* has been incorporated into Japanese modes of life, but retains its meaning as an air, vapour or steam that when surrounding a person can in certain situations be translated to ‘mood’. In this sense, *qi*/*ki* is an embodied wind of spiritual inspiration, inhalation and exhalation: a way of living and the movement of being directly aware. Elisabeth Hsu, writing of *qi* in Chinese medical texts explains that while *qi* represents regular wind/breath, *feng* means unruly wind (2007: S117). Mood, according to *qi* is awareness grasped intuitively and can affect or be affected by the winds or air, and hence ill mood has been described as *kinchosita kuhki*, or a tense air in Japanese, and *feng-*winds became the main aetiology of madness in Medieval China (Hsu, 2007: S117; Ogawa, 1998: 322). *Qi* thus, as mood, movement and atmosphere, ‘fills the body and flows over the world at the same time’ (Ogawa, 1998: 333).

In this regard, breathing and movement practices originating with concepts of *qi* (explored as *chi* in some detail in Gatt’s article, this issue). *Chi/qi*-based practices such as Qigong and Tai Chi, while incorporating different techniques and aims, focus on balancing mood, and regulating breath in a way similar to the wider goals of yogic practice: connecting mind, body and spirit to reach for enlightenment whist stabilizing health. Qigong, Tai Chi, yoga, meditation and new derivatives of mindfulness have been commended for their health benefits as complementary therapies to western biomedicine, including in applied research looking into anxiety reduction and improvement in lung function for those living with respiratory illness (e.g. Chan et al., 2011; Donesky-Cuenco et al., 2009; Ng et al., 2011; Pomidori et al., 2009; Yeh et al., 2010).

## Breath, Magic and States of Being

The idea that breath can be cultivated or manipulated to change states of being, whether to stabilize or support health, or influence undesirable experiences, has also been explored in relation to cross-cultural ethnographies of shamanism, magic and the taboo. Malinowski, for example, details how, for the Kula, magic is pivotal to ensure good luck for overseas expeditions. Breath for the Kula gains its significance through the breathing of spells and particularly during the process of building and preparing canoes for overseas departure. A few days before sailing, the *sulumwoya* (mint plant) spell is uttered the evening of the day canoe owners receive the gift of a pig or two from the expedition master. Moving a sprig of mint to and fro in their hand, canoe owners would chant this last verse of the *sulumwoya* spell:

Recently deceased spirit of my maternal uncle Mwoyalova, breathe thy spell over the head of Monikiniki. Breathe the spell upon the head of my light canoe. I shall kick the mountain; the mountain tilts over; the mountain subsides; the mountain opens up; the mountain jubilates; it topples over. I shall kula so as to make my canoe sink. I shall *kula* so as to make my outrigger go under. My fame is like thunder, my treading is like the roar of the flying witches. ([1922] 2014: 210)

Malinowski also offers a glimpse into the importance of breath in the performance of such spells. In *Coral Gardens and their Magic* ([1935] 2013), Malinowski notes that when the Trobriand Island magician enacts the spell into being, it is the action of the throat as the base of intelligence or mind that ‘imparts the virtue to the breath of the reciter’ which can then be transferred to the charmed objects (p. 445). In Hutton Webster’s study of magic, he compares this notion of power to understandings of magic for the Mala of the Solomon Islands and those from Malekula, in the New Hebrides. Mala believe that the seat of magic is held within the chest, and thus to release the spell, the magician must breathe hard or spit to ensure its strength (Webster, 1948: 116). For those living in Malekula, sorcerers gain their strength from holding their breath and at the end of exhalation the imprecation is chanted, releasing his vital emotion (ibid.).

How the technique of breathing links with the production of meaning in ritual and ceremony has also been explored throughout the rich accounts of shamanism and tobacco smoking in *The Master Plant: Tobacco in Lowland South America* (Russell and Rahman, 2015). Elizabeth Rahman writes of how the regulation of breathing in the practice of *prānāyāma* can be seen as similar to the inhalation of deep lungfuls of smoke by shamans; even if the sought vitality is attained through toxic fumes (2015: 146). Noting that smoking makes breath visible, Françoise Barbira Freedman further reveals that purposeful breathing techniques utilized by Keshwa shamans are tied to wider ontological forces:

Before blowing tobacco smoke and sucking out pathogens, shamans need to engage their breath through silent rhythmical whistled exhaltations (in rare cases inhalations) or as a soft voiced whistling that can vary with greater or lesser use of sinilants, fricatives or shh sounds. This whistling is a prelude to incantations, which are part of shaman’s internal store of knowledge. Culturally standardized combinations of vowels (*nay-nay*, *rin-rin*) may be sung in transition from whistling to songs (Q. *ikaro*) attributed to particular powerful plants or to spirit allies. The whistling is associated with the use of shakapa leaf rattles or to spirit allies. (2015: 77)

For the Keshwa shamans, the sucking in of tobacco fumes and blowing out of smoke appeals to all of the senses; variations of performance provide appropriate energy to the pipes and rattles that negotiate magic and thus become more than objects. They act as agentive ‘journeying instruments’ that assist in bringing about different modes of consciousness (p. 77). Calling into mind Merleau-Ponty’s analysis of the blind man’s cane ([1945] 2002: 165), these instruments project a vital intentionality that extends through the implements into the wider cosmos.

As intentional practices, specific ways of breathing are highly strategic; there are breaths that do good, and breaths that do ill. Synchronized, collective breathing through multiple instruments and particular movements can bring about trance-like, ecstatic states. Such corporealities can be experienced by Turkish Sufi brotherhoods through music, poetry, *sama’* (dance) and *dihkr/zikr* (breath training), by the Teimars of peninsular Malyasia through healing bamboo-playing and singing rituals, and by those who can ‘speak in tongues’ or experience glossolalia (Gore, 1995: 43; Markoff, 1995: 157; Roseman, 2007: S63). For Shipibo-Konibo and Suyá healers, the synchronization of breath through singing with blowing can permeate the sick with homeopathic properties. The bodies of the sick are metaphorically pierced or injected via song (not necessarily sung out loud) and as such provide another example of how the breath can become a tangible instrument of great power (Brabec de Mori, 2015: 27–28).

Another graphic illustration of the power accorded breath are the Nilotic languages in which the root for both ‘breath’ and ‘life’ are the same (*wei* in Shilluk and Dinka; *we* for the Lango of Uganda). *Wei* can also be translated as soul or spirit and is what distinguishes all things active and alive from what is inert and lifeless (Lienhardt, 1954: 155; Lienhardt, 1961: 206). As mentioned above, the significance of breath for the Dinka means their ritual clan chief cannot be allowed to die of ‘natural’ causes but must be suffocated (usually by being buried alive) so that the life force that is contained in his breath is able to permeate the whole community, rather than being lost. Similarly for the Nyakjusa of Tanganyika (now Tanzania) and Ngonde of Nyasaland (now Malawi), a king had to die ‘with the breath in his body lest the power of growth disappear with him’ (Wilson, 1959: 18). ‘Vitality, energy, physical vigour, force of character are all important, and must be guarded and fostered…energy was admired and judged to be, in a sense, divine’ (Wilson, 1959: 4).

a Nyakyusa chief was expected to show his vitality in dancing and hoeing, in the number of his children and the multiplying of his herds, in the success of his army, and his force of personality. He feared to lose vigour for that meant death: no people could afford to tolerate an ageing or ailing king. (Wilson, 1959: 4)

Chyme is a third substance equated to ‘life’ and ‘breath’ in the Dinka language, something which ‘makes the flesh and maintains the life of the beast…smeared on a Dinka at his adoption into a Nuer lineage’ (Evans-Pritchard, 1956: 214). For the Lango of Uganda, the life force ‘*we*’ enters a person at birth and is transmitted through the patrilineage. It is also breath and chyme, both of which emanate from the stomach, the vital centre of the body (Curley, 1973: 137).

Wilson (1959) extemporizes further on the ‘body social’ and ‘body politic’ implications of breath in Nyakyusa–Ngonde cosmology. The ‘breath of men’ in her title is the murmurings of discontent by commoners against a chief (the same word is used for the buzzing of a hive of bees). Anger is the root of such murmurings, which are regarded as the moral counterpart to immoral witchcraft.

In the Nyakyusa view the legitimate anger of a man’s neighbours, particularly of his village headman, may cause him to fall ill of a fever; as they put it, men murmur…and their cold breath falls upon him. (Wilson, 1959: 9)

Thus the ‘breath of men’ is used by the community to enforce law and morality. Confession and forgiveness are symbolized by spitting, or blowing out water.^3^

The act of blowing, however, for the Akawaio of Amazonia and Tsimshian Indians of British Columbia can signal great danger. Audrey Butt has described how blowing for the Akawaio can result in ‘blowing sickness’. An Akawaio shaman in séance can decipher three spirits in those ill from such sickness; the sick man’s own spirit, the spirit of the person who blew the sickness and a nature spirit summoned to the victim’s aid via specific incantations (1961: 148). For Tshimshian Indians, the blowing of the wind is linked to the action of human twins, and they thus implore to the wind and rain ‘Calm down, breath of the twins!’ (Frazer, [1922] 2000). Particular modes of breathing thus offer a means of exploring relationships between people, spirits and the wider environment, but breath as an active and agentive property can also act as a form of communication in itself.

## Breath and Work

While much of the research that touches on breath focuses on its correlation to life, death, healing and illness, the harnessing of breath or its experience can also be crucial to understanding work and being out of work in specific contexts. Brian Lande’s excellent article ‘breathing like a soldier: culture incarnate’ (2007) details how learning to breathe in an institutionalized way can form a process of initiation in a military setting; breath training can be a survival exercise as well as one which bonds soldier with soldier. For the *Ama* of Japan, or traditional spearfishing divers of Bajau, holding ones breath is the means for economic survival, and proficiency also provides a means of acceptance in their coastal communities (Nukada, 1965: 25; Schagatay and Abrahamsson, 2014). The breathing techniques and practices utilized by *Ama* and Bajau fishermen also have an interesting crossover into the world of competitive free diving. Such correlations between sport or leisure and work also offer a way to explore further how socio-economic contexts are embodied in the act of breathing. The *katajjaniq* breath/vocal game of the Inuit, for instance, manifested as a pastime for women awaiting those to come back from hunting trips. Similar to the *Rekkukara* of the Japanese *Ainu*, the game is played face to face in close proximity with women sometimes holding each other shoulders. The game stops when one of the women laughs or runs out of breath (Nattiez, 1983a, 1983b, [1987] 1990).^4^

Socio-economic factors have also been proven embedded in breathing activities such as smoking. While the context for smoking has changed over time, in the United Kingdom, current rates of smoking prevalence are higher in groups of lower socio-economic status (ASH, 2015). The links between work and breath have thus become particularly important when considering the histories of breath for people considered marginalized, and global health initiatives that seek to identify and work with groups that may be or have been more susceptible to particular breathing experiences. Acknowledgement of industrial exposure to fumes and substances in the air has become particularly prevalent in terms of examining lung disease that can occur from working with coal or asbestos. One of the most notable texts to arise from such discussion is Barbara Ellen Smith’s (1981) work on the West Virginian 1968 ‘black lung’ movement. Ellen raised awareness of interacting sociopolitical perspectives within this movement, namely opposing medical, occupational and cultural forces that sought to define the ‘black lung’ illness and how it should be recognized, treated and dealt with. She concluded that the ‘black lung’ was not an inevitable illness for miners, but one that was produced through various social relations. The implications of this in comprehending social understandings of disease were clear and remain relevant in researching illness today, such as when considering inhalant misuse, or ‘huffing’ or ‘sniffing’, that has been labelled a serious and ongoing problem in remote Aboriginal communities in Australia.

## The Papers in This Issue

The topic of interdisciplinarity weaves its way through all the articles in this issue, like it does (or should do) in breath studies and body studies more generally. It appears either as a central focus of Macnaughton and Garnett or as a provocative challenge to those working in other disciplines to take the subject matter of the article in question seriously (in the case of Allen, Gatt, and Rahman and Brabec de Mori). Jane Macnaughton’s article derives from the inherent interdisciplinarity of the medical humanities and uses the exemplar of the *Life of Breath* project to make its point as to the value of this approach. However, it also takes issue with other work in the medical humanities by its titular argument for a critical medical humanities (CMH) approach. For a long time championed as an educational tool for the creation of better, more humane doctors, in taking a critical stance, the medical humanities aims for transdisciplinary methods and to extend the healthcare evidence base. Furthermore, the CMH approach, as reflected in Macnaughton’s article, has a much wider range of clinical and nonclinical health and social care practitioners and their patients/clients within its reach. Macnaughton’s argument is that the invisibility of breath has not only individual, social and political but also linguistic manifestations. Furthermore, the lack of a non-clinical ‘language of breath’ results in the articulation (in Latour’s sense) of sensations of breathlessness being largely hijacked by clinically modulated interpretations. This has implications for how symptoms are experienced and expressed and the so-called symptom discordance in breathlessness. Neuroscience is starting to accept the importance of affect and cognition in understanding the relationship between body, brain and world and can assist us in understanding symptom perception. The role of ‘priors’ (a reductive term for the concept of previous experience) draws attention to the need to consider lived experience within scientific experiments that seek to provide data of relevance to people who suffer from chronic breathlessness. The development of interoceptive awareness for people in this situation, such as through free movement activities and dance, offers some hope for people seeking a measure of control over a singularly terrifying and debilitating symptom.

Interdisciplinarity is also a central element of Garnett’s article, but in a more practitioner-focussed sense, since it is based on embedded ethnographic research that aimed to understand how interdisciplinary knowledge about air pollution is achieved and practiced among a category of scientists known as ‘building physicists’ and the more familiar epidemiologists. The building physicists, through their computer model/simulation of indoor atmospheres, became aware that ‘breathing bodies’ challenge normal modelling because of their significant, but frequently unquantifiable and unpredictable effects on indoor air quality. Modelling ‘breathing spaces’ disrupted notions of bodily and environmental integrity that had initially underpinned the study. Actions like ‘window opening’ were disruptive, not only for the effects of window opening on air flow rates in domestic environments but also for how particles inside the building were affected. What she calls the ‘respiratory dynamics’ of buildings emphasizes the blurring of bodies and environments in ways that are problematic to traditional means of understanding human exposure to air pollution.

Body–air relations of a different sort are to the fore in Allen’s feminist political ecology of air-and-breathing-bodies. This is characterized by an attention to the immaterial and invisible, intimate scales of breath, the intersectional aspects of corporeality and lived experience/embodiment of air-and-breath with its relational, emotional and affective dimensions. Allen points out that despite a plethora of apolitical medical, technical and environmental health literature, air has been much less evident than water in the political ecological canon. Even where urban airs (for example) have been the subject of political ecology, the living, breathing body is still only an implicit presence, and air and breath as non-human, agentic entities remain shadowy at best, perhaps due, Allen suggests, to a masculinist ‘logic of solids’. Conceptions of an ecological body shift into new breathy-airy body spaces when a political ecology of air is brought together with a feminist political ecology of bodies; breathing becomes a form of knowing, and air pollution and dust are framed as silent, violent aggressors running counter to the affective and aesthetic dimensions of a ‘poetics of air’.

Feminist political ecology avoids the potential depoliticization incurred through a meaning-less focus on affect and posthuman relational ontologies. This is due to its simultaneous focus on structural, post-structural, material and discursive domains as well as through body studies’ investigations of the ontological and epistemological implications of a revitalized corporeality. From Allen’s perspective, the emotional, affective and atmospheric encounters that shape political ecological claims and experience have gone largely ignored.

The last two papers operationalize some of these theoretical and experiential tropes in two highly contrasting locations – A Grotowskian laboratory theatre, and two Amerindian communities, the Warekena of the northwestern Rio Negro, Brazil and the Shipibo-Konibo of the Ucayali valley in eastern Peru. Caroline Gatt describes the linguistic and musical qualities of voiced breath and the challenges it provides to theoretical notions of ‘embodiment’ in theatre/practice studies. In much performance practice and theory, the body, affect and movement are regarded as ontologically primary, and language, reflection and semantic analysis seen as secondary. Spontaneity, somehow, is tarnished by conscious, linguistic or referential thought. Gatt argues against such a view through an ethnographic account of meeting her Maltese grandmother through voiced breath (in this case, a song that maintains some of the qualities of Grotowski’s ‘vibratory song’). In this way, she suggests, laboratory theatre becomes an ‘art of enquiry’ offering a means of accessing and engaging with traces from the past that are ill-apparent in more materialist conceptions of history. In this way, her article is an exegesis of a new form of disciplinary endeavour and emphasizes the importance of tackling the ephemeral, unproven and eerie in our historical analyses.

For the Amerindians in Rahman and Brabec de Mori’s account, smoking is added to singing as the two most life-enhancing ways of reconfiguring breath, ‘that potent evidence of human vitality’. In both cases, breath becomes the interface between human and non-human subjects and subjectivities, a means for specialists to manage their vitality in ways that produce potent effects both in themselves and the world around. As with the laboratory theatre example, the voice is intrinsically linked to breathing, but also to blowing, smoke and other visual and olfactory senses. In this case, breath is the active subject of regulation and control, the source of ritual skills performed by those regarded as having attained the highest levels of mastery over their own and others’ noncorporeal human vitalities. Air, wind, smell, sound, aura, atmosphere and breath are all synonyms encompassed by the Shipibo term *niwe*, the creation and management of which are of paramount ritual intent. Bodily senses like sight, touch and taste only perceive the ‘tunic of things’, whereas *niwe* is ethereal, invisible and extends through and across the material world of worlds in shared, interaffective ways. Like air, wind and breath, *niwe* is a vital force that flows in, out and between people and the world.

## Conclusion

Breath is an inherently interdisciplinary topic, encompassing cutting-edge research in both clinical/scientific and non-clinical contexts. Prior work in this field is relatively sparse and highlights the importance of unpicking the significance of breath in specific contextual ontologies and epistemologies. Breath addresses issues in conventional social research areas such as life, death, health, illness, work and leisure. The articles in this special issue introduce breath as a cosmological entity, a temporal marker and a performative technique embodied into everyday lived experience. They set a waymark for further work in the emergent field of breath and body studies. Some areas remain untouched by these contributions. We have not delved into in-depth experience with breathing apparatus, for example, or the wealth of work on public respiratory health engagement. Instead, they offer a means to ‘denaturalise the given’ (in the words of Margaret Lock and Judith Farquar 2007: 212), noting that it is through questioning the essence of breath, that we might begin to comprehend just what breath *is*, and what it means to live and breathe. Providing context for what otherwise has been left unseen, unelaborated or taken for granted, it offers a way to begin to make the invisible, visible.

## Notes

1. It is worth noting, in our focus on breath, that such epistemologies are not always central to modes of being. The Wola of Papua New-Guinea, for example, regard the liver as much more important than the lungs, and blood as more significant than breath (Sillitoe (1986), personal communication).

2.
Parkin (2007) notes that the semantic template includes: …the Sanskrit *aniti* (he breathes, that which is animate) based on the root *an* (to breathe), and hence *pra-(a)na(m)* (life), and *atma(n)* (soul); Greek *anemos* (wind), *pneuma* (air, gaseousness); Latin *an-imare* (to give life to), *anima* (breath, soul), *animus* (spirit)…. [This template is echoed in] Arabic-Jewish *nafesh* or equivalent ranging over soul, life, person, living being, blood, desire, and, related, breath and sweet odour, and with *neshama* and *ruakh* also shading firmly from ‘breathing’ to the idea of ‘spirit’. (S40)
Parkin then discusses the Bantu term *Pepo*, noting that this term ‘is part of a polythetic range in the region meaning air, wind, cold, spirit (e.g. Giriama *peho*, cold (air), wind, *pepo*, spirit; Nyakyusa *imbepo*, air, wind), and, as well as carrying such connotations, has even been harnessed by Muslim Swahili speakers to refer to Paradise (*Upepo*, i.e. the great spiritual air above)’ (S41).

3. For the Shilluk, water is eternal whereas fire is ‘like blood, the stuff of worldly transformation…Fire meets water as mortal man meets god’ (Graeber, 2011: 40)

4. There are many other examples of how technical breathing skills links work with leisure and enjoyment, particularly in terms of singing and theatre performance. Sreenath Nair, in his work *Restoration of Breath: Consciousness and Performance* (2007) describes actor training in Eastern forms of theatre. Focussing on the *Rasa,* he correlates the expression of emotion with particular breathing patterns. For example to portray ‘eroticism’ means ‘taking breath down from the lower abdomen and pushing it slowly and gradually up until it reaches at the middle centre of the head’ (p. 127).
